# Diet, lifestyle, and livelihoods during coronavirus disease 2019 (COVID-19)–related lockdowns and the value of web-based nutrition studies

**DOI:** 10.1093/ajcn/nqaa408

**Published:** 2021-02-10

**Authors:** Anika Knuppel

**Affiliations:** Cancer Epidemiology Unit, Nuffield Department of Population Health, University of Oxford, Oxford, United Kingdom

See corresponding article on pages 924 and 984.

See corresponding articles on pages XXX-XXX and XXX-XXX.

In October 2020, the WHO warned of the impact of coronavirus disease 2019 (COVID-19) on nutritional status and quality through changes in socioeconomic conditions (1). Although these predictions were mainly speculative at the time, 2 articles in this issue of the Journal now add prospective evidence on changes in diet quality and lifestyle during the April 2020 COVID-19 lockdowns in relation to sociodemographic circumstances. Both studies used data from ongoing web-based cohort studies, NutriNet-Santé from France and NutriQuébec from Canada, which allowed them to conduct this timely research (2, 3).

Deschasaux-Tanguy et al. (2) surveyed a subsample of NutriNet-Santé participants and compared their diet quality from April 2017 to 2019 with April 2020; the results showed no changes in overall diet quality (based on the Alternative Healthy Eating Index), but decreased total energy, carbohydrate, fish, vitamin B-12, and protein intakes. Using a questionnaire on lifestyle change perceptions, the authors also found 3 clusters of change: 42.9% of participants experienced no changes; 37.4% experienced unfavorable changes such as increases in weight, sedentary time, and sweets consumption; and 19.8% experienced favorable changes including increases in fresh fruit, vegetable, and fish consumption, and adapting food and drink choices for weight-management purposes (2).

Lamarche et al. (3) compared diet quality in April–May 2020 with that in June 2019–February 2020 in a subsample of NutriQuébec participants. The authors found a small increase in overall diet quality compared with baseline (+1.1 points; 95% CI: 0.6, 1.5 points; based on the Healthy Eating Index–2015, which ranges from 0 to 100 points), with increases in whole grains and reductions in added sugar. However, there were also reductions in fruit intake, increased sodium intakes, and unfavorable fatty acid ratio changes. In contrast to the French study, NutriQuébec was unable to clarify whether associations were the result of the lockdown or seasonal variations. The other main outcome reported was food security, which increased from 96.2% to 99.0% during lockdown. However, this was based on very few participants reporting food insecurity at baseline (*n* = 26), which increases the risk of a chance finding, and the authors clarify that mitigating measures were implemented in Quebec at the time of lockdown (3).

In addition to describing changes in diet and lifestyle, the 2 studies also found associations between these changes and sociodemographic conditions. For instance, participants in the “no change” cluster, identified in Deschasaux-Tanguy et al. (2), had no changes in professional activity throughout lockdown, but both the “favorable” and “unfavorable” clusters experienced professional activity changes, such as working from home full-time or not working. The “unfavorable change” cluster was more common among women and associated with lower income but higher education, working full-time from home, and being more likely to have children aged <18 y in the home. Previous research has shown that women who are homemakers have a higher risk of obesity than those working in a steady job (4) and that informal caring responsibilities alongside working full-time are associated with higher adiposity in young women (<44 y) (5).

The “favorable change” cluster was associated with having a higher educational level, higher income, and working from home with no children aged <18 y in the house. This group was also more likely to be in the overweight or obese category (2), which was in line with the unadjusted results from NutriQuébec that found larger diet-quality improvements in those with obesity (3). Further research is needed to investigate whether this could be related to reduced exposure to an obesogenic environment, such as out-of-home eating, which has been shown to be associated with weight gain (6). In NutriNet-Santé, 40.4% reported no longer eating out (2), and in NutriQuébec, the proportion of meals consumed outside the home decreased from 21.2% to 3.6% (3).

Both studies demonstrate the value of web-based nutrition studies. The web-based design allowed the researchers to add COVID-19–related questionnaires at short notice and to quickly collect dietary data. Traditionally, 24-h dietary recalls were considered expensive and time-consuming, requiring trained interviewers to conduct in-person or telephone interviews, ideally on several occasions per person (7). Using web-based versions of 24-h dietary recalls requires fewer resources and simplifies repeated measurements.

A major limitation of both studies is the risk of selection bias and likely limited generalizability. Both studies collected data from volunteers; therefore, there was likely self-selection bias in the baseline sample, with participants being overall healthier and less disadvantaged than their source populations. Furthermore, additional self-selection bias could have incurred because of the low response rates of those who answered the additional questionnaires during lockdown [24.5% in NutriNet-Santé (2) and 37.0% in NutriQuébec (3)]. This is especially concerning when investigating associations with socioeconomic measures as the most disadvantaged groups will be missing from the samples. For example, the prevalence of food insecurity, which the Canadian Community Health Survey reports at 8.5% in Quebec (8), was ∼4 times the prevalence in the NutriQuébec subsample (2.2%) and nearly double the prevalence in those who did not respond (4.8%) (3). Additionally, web-based studies require internet access, which could inherently exclude whole parts of the population. Even in high-income countries internet access has been shown to be low in older individuals (≥55 y) with low educational levels (e.g., 48.8% in France) (9). Both studies used weighting to account for selection bias, but it is unlikely to eliminate it, also because socioeconomic characteristics at follow-up, such as a job losses or major income changes, could not be accounted for.

In summary, the research by Deschasaux-Tanguy et al. (2) and Lamarche et al. (3) suggests that diet quality and lifestyle changes during the first COVID-19 lockdown in April 2020 were complex and may depend on sociodemographic conditions. These findings were made possible by the web-based design of these studies. However, future online studies need to ensure access and participation of disadvantaged groups, as well as retain participants over follow-up. With ongoing changes such as changes in food acquisition, income, and working environment in response to COVID-19 (1), web-based (nutritional) cohorts will become a rich resource to produce novel and timely evidence.

## Notes

The author is supported by the Wellcome Trust, Our Planet Our Health (Livestock, Environment and People - LEAP, 205212/Z/16/Z).
